# Sample preparation under turbulent flow with renewable sorbent†

**DOI:** 10.1039/d1ja00252j

**Published:** 2021-10-07

**Authors:** David J. Cocovi-Solberg, Stephan Schnidrig, Stephan Hann

**Affiliations:** University of Natural Resources and Life Sciences, Vienna Muthgasse 18 1190 Vienna Austria david.cocovi-solberg@boku.ac.at +43 1 47654 77109; VICI AG International Parkstrasse 2 CH-6214 Schenkon Switzerland

## Abstract

Turbulent flow chromatography is an online solid phase extraction mode that achieves the extraordinary effect of proxying an upper molecular weight cutoff for the retained molecules, based on loading the sample at high linear velocities. Despite the potential of being a universal sample preparation technique prior to inductively coupled plasma mass spectrometry and liquid chromatography mass spectrometry, it employs specific hardware and expensive consumables. In the present work we apply this technique using off-the-shelf fluidic components and the niche “bead injection” methodology. For the first time, this procedure has been executed with a pressure of approximately 20 bar, compared to the low pressure of the classic setup, achieving a sample throughput >285 h^−1^ for the SPE/TFC procedure, or 20 h^−1^ if the procedure involves renewing the sorbent, using no more than 4 mg of sorbent for every μ-SPE. Another novelty is that sorbent packing and unpacking has been controlled with a smart method using real-time pressure feedback as quality control for truly unattended operation. Finally, the turbulent flow chromatography principle has been comprehensively characterized, providing similar performance to that demonstrated in earlier literature, and the ancillary sample preparation capabilities, *e.g.*, in-valve acidification, have been demonstrated by the fractionation of gadolinium in surface waters prior to ICP-MS, an element of increasing surface water concern due to its use as a magnetic resonance contrast agent.

## Abstract 2

An open platform for sample preparation under turbulent flow conditions with stationary phase renewal and smart control.

## Graphical abstract

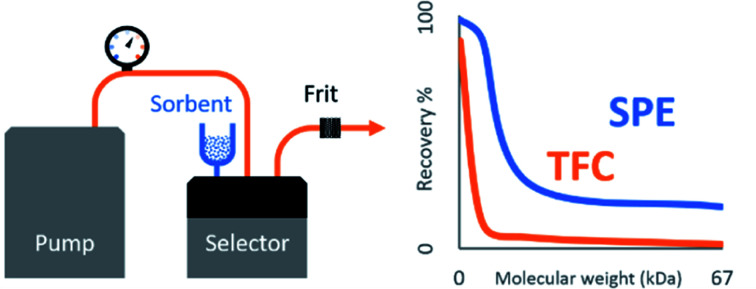

## 1. Introduction

Turbulent flow chromatography (TFC)^1,2^ is a solid phase extraction (SPE) mode patented and commercialized as a universal sample preparation platform for clinical and environmental analysis. It is based on the differential mass transfer of low- (LMWC) and high-molecular weight compounds (HMWC) when loading a packed SPE column at high linear velocities: the LMWC enter the pores because their transport is diffusion-controlled, but the HMWC are excluded from the sorbent material and exit the column without interaction because their transport is dominated by advection.^1,3–6^ Apart from the SPE benefits, such as enrichment of target analytes, matrix cleanup and medium exchange to name a few, this SPE mode introduces a molecular weight cutoff, that is, the HMWC are not retained, allowing a much cleaner downstream analysis, and are extremely fast due to the high linear speeds used. For these reasons, in clinical or environmental analysis, it is desirable to use TFC. Regretfully, the hardware for TFC is dedicated, and the TFC columns are expensive consumables of non-disclosed chemistry and a limited lifetime, since their performance decreases due to irreversible sorption of matrix components and compaction, as happens for all online cartridges. Accordingly, the long-term unsupervised operation of this powerful technique demands a way of renewing the stationary phase while minimizing the hardware requirements.

Bead Injection (BI)^7–9^ is a fluidic technique introduced in the early 90's that aimed to automate the SPE procedures including the sorbent renewal. In that approach, SPE sorbents are suspended as slurries and manipulated by flow programing in a closed manifold built around a stream selector and a bidirectional pump. When the sorbent slurry is perfused through an inline frit, the liquids flow through, but the sorbent compacts, forming an inline μ-SPE column. The main benefits are the reduced use of sorbent, which is in bulk form and excludes the use of plastic cartridges and the renovation at will of the sorbent phase, which is of interest to us for automating the TFC. However, the irreproducibility in packing and unpacking the sorbent prevented a truly unsupervised operation that disputed its success. We also integrated a smart control of the packing and unpacking of the sorbent using the pressure feedback as a QC for truly unsupervised operation. Incidentally, the particle sizes, inner diameters, sorbent chemistries and column-to-particle ratios are the same in BI and TFC. To the best of our knowledge, BI has never been used as a platform for TFC, because the first employs 5 bar capable syringe pumps, while the latter is used with HPLC pumps at approximately 30 bar. The commercialization of new medium pressure models did not change the above-described landscape until now.

In the present communication we assembled a sample preparation system making use of the above-mentioned concepts, and as a proof of concept in the frame of environmental analysis, we have fractionated gadolinium in surface waters using an elaborate method prior to ICP-MS detection. Gd is a trace element of increasing environmental concern. Its use as a magnetic resonance imaging contrast agent and the impossibility of retaining it in wastewater treatment plants leads to an increase of its surface water concentrations. The aim of this contribution is to demonstrate the feasibility of implementing TFC with BI hardware for automatic solid phase renewal, and to control the system with a smart method for improving the reproducibility of BI and allowing a truly unattended operation.

## 2. Experimental

### 2.1. Reagents, sorbents and samples

Gd and In standards were prepared by successive dilutions from elemental Gd and In standards of 1 g L^−1^ in 2–3% HNO_3_ (Merck and Inorganic Ventures, respectively). Ultrapur 60% HNO_3_ from MERCK was used after subboiling (DuoPUR subboiling distillation system, MLS, Sorisole, Italy). Milli-Q water (*ρ* > 18.2 MΩ cm) was used for preparing all the solutions and as a carrier for the fluidic system. Gd standards were buffered in 20 mmol L^−1^ NH_4_AcO at pH = 6.5 prepared from anhydrous acetic acid (Merck) and 25% NH_4_OH puriss (Sigma-Aldrich).

An Oasis MCX 60 μm mixed-mode strong cation exchanger-reversed phase sorbent (Waters Corporation, Milford, MA, USA) was used as a sorbent for the preconcentration of Gd. The advantages of this sorbent are its broad availability, spherical shape and narrow size distribution that allow an easy fluidic manipulation. Also, its polymeric nature compared to hard silica makes it less prone to scratch the surfaces of the fluidic components. Its particle diameter, internal porosity and polymeric nature match the solid phases of commercial TFC columns. The sorbent was dispersed in methanol for manipulating it in the manifold. The density of the dry beads was 1.10 g mL^−1^, the density of the methanolic slurry 0.965 g mL^−1^, and the concentration (w/w) 0.44%.

Human serum albumin, myoglobin from horse skeletal muscle and insulin from bovine pancreas (all from Sigma Aldrich) were selected for characterizing the TFC performance because their molecular weight distribution (66.4, 17.8 and 5.7 kDa, respectively) allows a reasonable coverage of potential interference in clinical or environmental analysis. Solutions were prepared in 5 mmol L^−1^ NH_4_AcO at pH = 4.75, near or below the isoelectric points of the proteins (4.7, 7.2 and 5.3), fostering both electrostatic and reversed phase interactions with the sorbent. The same buffer was used as a carrier in the TFC experiments.

Water from an algae-bloomed pond influenced by the Danube river was filtered through a 0.45 μm cellulose filter and used as a worst-case model sample due to the high organic matter content.

### 2.2. Instrumentation

**Fig. 1:**
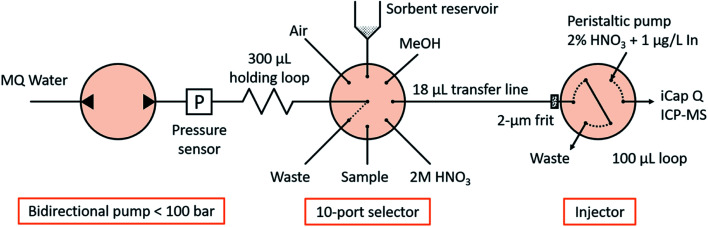
Scheme of the fluidic manifold.

The fluidic manifold (Fig. 1) consists of a bidirectional pump (<100 bar, model M6HP), a 10-position selector with a 0.4 mm feature size and an injector, all of them provided by VICI AG International. The holding loop, transfer line and injection loop were made of PEEK of 0.5 mm i.d. and 1/16′′ o.d., and had volumes of 300, 18 and 100 μL, respectively. Those diameters were selected for minimizing the tortuosity in the fluidic connections and easing the sorbent manipulation, as well as for matching the dimensions of commercial TFC columns (see Section 2.4 ‘Characterization of turbulent flow chromatography’). All other tubes in the system were FEP tubes of 0.25 mm i.d. and 1/16′′ o.d. with a length of approximately 20 cm, except the connection to waste, with 1 mm i.d. to allow the unconstrained disposal or recovery of the sorbent. A titanium frit of 1/16′′ o.d., 0.040′′ thickness and 2 μm nominal pore size was introduced in the injector pilot of the transfer line to retain the sorbent (see Section 2.3 ‘Sorbent manipulation and smart control’). The sorbent slurry was held in a 1 mL polypropylene syringe barrel, coupled with the selector in an upright position *via* an adapter. A LiVi-Ti-01-5000 biocompatible (titanium-wetted) pressure sensor from DJ Instruments (Billerica, MA, USA) was inserted between the pump and the holding loop; the signal was amplified 5 times and read through the ADC pin of the pump actuator with a resolution of 0.070 bar and a full scale of 0 to 77.22 bar.

An iCap Q ICP-MS system controlled by Qtegra software (Thermo Fischer Scientific) was used as a detector. The carrier was 2% HNO_3_ at 100 μL min^−1^ containing 1 μg L^−1^ In as an internal standard controlling the injection procedure. The nebulizer was a PFA microflow nebulizer from Elemental Scientific (Omaha, Nebraska, USA), and the quartz glass cyclonic spray chamber was kept at 2.7 °C with Peltier cooling. The nebulizer gas flow rate was 1.02 L min^−1^ and plasma power 1550 W. Intensities of ^158^Gd, ^115^In and ^32^S^16^O (*m*/*z* = 48) were monitored in oxygen reaction mode (0.338 mL min^−1^) and acquired with a dwell time of 0.1 s and 0.1 s spacing. The interference of the titanium frit and pressure sensor (also detectable at *m*/*z* = 48) was studied and proved to be negligible. Under those conditions, peak widths at medium height were approx. 40 s and were monitored during a total of 100 s upon contact closure after the injector moved to the inject position.

The freeware CocoSoft 6.1 ^10,11^ controlled all fluidic actuators, compensated the pump backlash, triggered the detector start through contact closure and acquired the pressure measurements for real-time smart control (see Section 2.3 ‘Sorbent manipulation and smart control’).

### 2.3. Sorbent manipulation and smart control

Bead injection,^12^ that is, the manipulation of SPE sorbents as slurries in fluidic manifolds, is a niche but established technique well within the capabilities of those skilled in the art. One of the novelties presented in this work is to implement it under smart control conditions to allow truly unsupervised operation. The volumes and flow rates herein presented are introduced as variables in the software, so they can be tuned manually or controlled programmatically in *e.g.* the optimization experiments, which cannot be done with the commercial alternatives. An annotated example of the method used during this work is presented in Table SI1.†

The method starts with aspirating 20 μL of methanol at 1 mL min^−1^, followed by 4 mg of sorbent into the holding loop. The method converts the desired sorbent mass into slurry volume by considering its weight fraction and density (see Table SI1†). The content of the holding loop is then dispensed to the transfer line: the beads are retained by the in-pilot frit forming a μ-SPE column, and the methanol and extra carrier flow towards the waste through the injector, cleaning and conditioning the sorbent. At this point the column is perfused with 300 μL Milli-Q water at 1 mL min^−1^ to compact the beads and measure the backpressure. If the pressure is lower than expected, the packing procedure is repeated for the calculated mass difference until the desired sorbent amount is successfully packed, that is, the same procedure is repeated in a proportional manner: additional mass = desired mass − currently loaded mass ∝ desired pressure − current pressure.

5 μL of sample were aspirated in the holding loop, followed by an “acceleration compensation volume” (see below) and dispensed through the sorbent by flow reversal, followed by 20 μL of additional carrier for washing. The dispensing flow rate was 1 mL min^−1^ if not otherwise stated, because this value corresponds to the same linear velocity utilized for provoking turbulence in commercial TFC columns with a similar particle diameter (60 μm), external porosity (40%), pore diameter (80 Å) and column-to-particle diameter ratio (8.3).^1^

The fluid inertia at the default pump acceleration provoked irreproducible injections that were solved by decreasing it from 1480 mL min^−2^ to 14.8 mL min^−2^. For preventing contact between the sample and the sorbent during the acceleration step, an extra carrier segment was aspirated after the sample. This acceleration compensation volume (in μL) was aspirated automatically without user intervention and calculated at real time from the kinematic equations as ∫*f*d*t* = *a*∫*t*d*t* = *at*^2^/2 = *f*^2^/(2*a*), where ‘*f*’ is the desired flow rate (μL min^−1^) and ‘*a*’ is the acceleration (μL min^−2^), allowing an easy variation of the flow rate or the acceleration.

After the sample loading and with the injector in the ‘load’ position, 20 μL of 2 mol L^−1^ HNO_3_ were perfused through the column for eluting the analytes,^13^ followed by 70 μL of carrier used to park the eluate in the injection loop. Finally, the injector was turned to the ‘inject’ position and the contact closure signal was sent to the iCap Q ICP-MS for starting the data acquisition.

An unpacking routine was created for discharging the used sorbent, consisting of rewetting it with 40 μL of methanol at 1 mL min^−1^ and re-aspirating it to the holding loop with a train of 5 pulses of 2 μL (for uncompacting), followed by the aspiration of 50 μL of solvent, including the sorbent, and discharge (or recovery) through the waste port. This routine can be called at will, *e.g.* after a given number of samples processed or upon decrease of the signal of the internal standard, and will be repeated until the pressure reading corresponds to the empty tube.

### 2.4. Characterization of turbulent flow chromatography

The exclusion molecular weight cut-off of the sorbent at different flow rates was characterized and compared to the performance reports of the commercial counterparts. 5 μL of three different solutions each containing 250 mg L^−1^ of one of the proteins (human serum albumin, myoglobin or insulin) and also containing 250 μg L^−1^ Gd were loaded onto 4 mg of MCX sorbent at flow rates from 50 to 2000 μL min^−1^. The same packed column was used throughout all the measurements to prevent artifacts from renewing, but the different flow rates were assayed in triplicate and randomized as QC against saturation or irreversible sorption. The non-retained fraction was trapped in the injection loop and injected for quantifying the non-retained fraction. After transfer to the detector, the valve was reset to the load position and the trapped compounds were eluted and parked with 90 μL of 2 mol L^−1^ HNO_3_ at 400 μL min^−1^. After the injection, 110 μL of 2 mol L^−1^ HNO_3_ and 200 μL of methanol cleaned the column from the sorbed proteins at 100 μL min^−1^ followed by extra carrier for reconditioning. To calculate the percentage of retention, the total sulfur and gadolinium contents were determined. The maximum sulfur content was calculated with a direct injection (same procedure without sorbent in the transfer line). Since no gadolinium was found in the non-retained fraction at 50 μL min^−1^, the maximum gadolinium content was selected as the amount present in the 2 mol L^−1^ HNO_3_ eluate at this flow rate.

### 2.5. Matrix cleanup

To assess the matrix cleanup capabilities of the loading under TFC conditions, a 100 ng L^−1^ Gd standard was prepared in a surface water matrix with a high organic matter content and acidified until pH = 2 with 2% HNO_3_. 1 mL of this sample was analyzed under SPE and TFC conditions (100 and 1000 μL min^−1^, respectively).

### 2.6. Proof of concept: application to natural samples

To demonstrate the sample preparation capabilities of the fluidic platform, a surface water sample with high organic matter content was processed in three different ways to speciate the present Gd, which was expected to be in the upper limit of reported anthropogenic concentration, *i.e.* above 100 ng L^−1^.^14^ In all cases 1 mL of sample was processed, eluted into 70 μL of 2 M HNO_3_ (preconcentration factor of 14) and injected in the ICP-MS, with the method shown in Table SI1.†

(1) SPE: the method preconcentrated 1 mL of sample at 100 μL min^−1^ (SPE conditions), with a total sample processing of 15 min 32 s. Those conditions will retain the free, namely aquo- or labile complexed Gd, as well as that bound to HMWC through cation exchange or reversed phase interactions. Early experiments (not shown) demonstrate that pharmaceutical formulations of Gd are not retained under those conditions.

(2) TFC: the same procedure was repeated under TFC conditions (1 mL min^−1^), with a total sample processing time of 4 min 36 s. High molecular weight interference will be excluded and only the free Gd will be determined. The difference between this TFC and the previous SPE method corresponds to the Gd bound to high molecular weight compounds.

(3) TFC under acidic conditions: the TFC procedure was repeated after acidifying the sample. Total Gd was thus determined, since the complexed Gd would be dissociated and determined as free Gd ions. The acidification was performed in one of the ports of the valve to which a 1 mL syringe body was connected. The sample was aspirated sequentially in aliquots of 100 μL, bracketed with 6 μL of 6 mol L^−1^ HNO_3_ and parked in that port during the SPE and TFC analysis (total of *ca.* 20 min). This interleaving gave enough time for the dissociation reaction to take place and made the overall method time efficient. Then this processed sample was analyzed with the TFC method. The difference between this AC and the SPE method corresponds to the Gd bound to neutral low-molecular weight compounds.

## 3. Results and discussion

### 3.1. Sorbent manipulation and smart control

According to Darcy's law and under the conditions of the hardware herein presented, the pressure measurement depends exclusively on the packed sorbent amount, because its flow resistance is significantly higher than those of the empty tubes. The sorbent was packed and the pressure measured. Afterwards the sorbent was recovered and the pressure measured again. The difference of pressure was correlated with the recovered beads according to pressure (bar) = 2.7187 loaded_mass (mg) + 0.1532, with *R*^2^ = 0.9657.

The proportional packing procedure required one or a maximum of two iterations for packing the sorbent with a total time of 58 s. A separate method routine was created for priming the sorbent channel upon sorbent exchange because in this case the smart method could take up to 10 attempts for achieving the desired backpressure, since the valve features were empty.

The unpacking procedure worked unattended in all cases, usually at the first attempt with a duration of 86 s. In a few cases, the pressure after the first iteration was close to the iteration threshold, *ca.* 1.2 bar *vs.* 1 bar and a second iteration was triggered. One reason can be that a small mass of sorbent (calculated to be >0.07 mg) was still present in the tube, probably pressed into the pores of the frit. Seldom when loading >1 mL of high organic matter content samples at flow rates of 2 mL min^−1^, the unpacking method was iterated up to 10 times. Early iterations did not decrease the pressure significantly until a given iteration unpacked the beads completely. This suggests that colloidal matter of the sample was deposited in the first beads forming a porous stopper, especially when compressed at 20 bar. Solutions could pass, but the beads were not free to move. We are currently designing alternative manifolds that allow the column to be unpacked using positive pressure on behalf of an increased sample throughput. Nevertheless, we consider the possibility of truly unattended operation and synchronization with the detector a significant improvement in the robustness and reliability of the bead injection methodology, and hope that the smart control herein presented can trigger its revival.

With a flow rate of 1 mL min^−1^ and a sample volume of 5 μL, all of the sample preparation takes place in 12.6 s: bracketing, aspiration of the sample, compensation of acceleration steps, adsorption of the analytes on the solid support and exclusion of high molecular weight interference, elution and parking. Neglecting other analytical steps that cannot be interleaved due to a longer duration such as chromatography or data analysis, this system provides a sample throughput >285 h^−1^, one of its main features. If the sorbent must be renewed for any new sample, the sample throughput decreases to 23 h^−1^, which is low compared to standard ICP-MS workflows, but much faster than LC-MS workflows. The analytical instruments' manufacturers could implement in their control software the possibility of selecting the loading flow rate, so end users could themselves implement the TFC principle in the autosampler when using a trapping cartridge of big particle size. Due to their motor power and plunger section, any modern autosampler can apply the required pressures.

### 3.2. Characterization of the turbulent flow chromatography

**Fig. 2:**
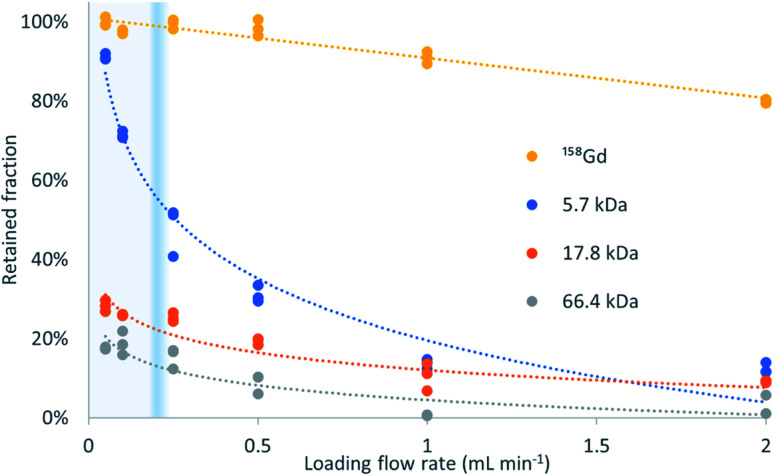
Retained targets of different molecular weights after loading on a μ-SPE column at different flow rates. LMWC diffuse fast and bind to the sorbent, while HMWC leave the column without interaction according to the TFC principle. The heavier the analyte is, the faster its recovery decreases when increasing the loading flow rate. The exclusion flow rate is molecular weight-dependent and the transitional flow rate from laminar to turbulent is marked in blue.

Fig. 2 shows the retained fraction against the loading flow rate, for the different molecular weight analytes (trends). It is calculated as the mass of analyte bound to the sorbent divided by the total mass of analyte injected, and expressed in percentage. A 2-way ANOVA found no significant differences between the gadolinium recoveries for the different mixtures with proteins (*p* = 0.247) but for the different loading flow rates (*p* = 3.13 × 10^−21^).

The percentage of exclusion against loading flow rate obtained in this experiment is in the same magnitude as data in pivotal papers of the TFC technique.^1^ The high recovery of Gd and negligible presence in the ‘non-retained’ fraction proves that its binding to the three proteins herein used is negligible at pH = 4.75.

The profiles obtained follow the expected behavior, that is, when the molecular weight increases, the retention decreases, and this decrease is more pronounced at high loading flow rates. The transition between >80% retention to <20% retention is very steep and allows a virtual molecular weight cutoff to be defined. In this example, the Gd retention decreases slightly with increasing flow rate but the recovery remains above 90% at 1 mL min^−1^. In contrast, the retention for the interference of molecular weights higher than 5.7 kDa is below 15%. The retention decreases at any flow rate with increasing molecular weight, being, at 50 μL min^−1^ and 2 mL min^−1^, 18% and 2%, respectively, for HSA (66.4 kDa), followed by 28% and 5% for myoglobin (17.8 kDa), and 91% and 12% for insulin (5.7 kDa). To this end, insulin perfectly exemplifies the above-mentioned principle and the successful implementation of the turbulent flow process. The behavior of myoglobin and HSA agrees with the data provided in the literature^1^ that suggest an absolute and flow rate-independent molecular weight cut-off of *ca.* 17 kDa.

Turbulent flow conditions are assessed in packed beads with the Reynolds number calculated as Re = *ρfD*(*Aμε*)^−1^, where *ρ* and *μ* are the density (g mL^−1^) and dynamic viscosity (g cm^−1^ s^−1^) of the mobile phase, *D* is the inner diameter of the tube (cm), *f* is the flow rate (mL s^−1^), *A* is the internal cross-section of the tube (cm^2^) and *ε* is the porosity of the packed bed (unitless), considering the laminar to turbulent transition at Re = 10 because of the high surface to free path ratio compared to open channels.^15^ In this work, the calculated Reynolds number accounts for the experimental porosity, and for 100 and 1000 μL min^−1^ it is Re_100_ = 2.41 and Re_1000_ = 24.1, indicating laminar and turbulent flow, respectively. Even supposing compaction to a theoretical minimum porosity (close-packing) of 25.95% the calculation yields Re_100_ = 4.09 and Re_1000_ = 40.9, suggesting those flow rates as good representatives of SPE and TFC conditions, respectively.

The theory behind the TFC principle states that the molecular weight cutoff effect is not determined by the classical Reynolds number, but for the ratio between advective and diffusive transport,^16^ which in our system corresponds to the reduced velocity or massive Péclet number, Pe = *udD*^−1^, which considers the linear velocity (*u*), particle diameter (*d*) and diffusivity of the different analytes (*D*). An analyte would experience the TFC effect when its Pe is greater than 5000.^16^Table 1 presents the Pe for the different analytes at different flow rates, proving again that the turbulent flow effect is not observed at 100 μL min^−1^, but at 1 mL min^−1^, with a diffusivity-dependent transitional flow rate.

**Table 1:** Calculated Péclet number for the different targets loaded at different flow rates. The bold type highlights turbulent effect conditions (Pe > 5000), that is, advective transport is more important than diffusive transport. The right column shows the transitional flow rate for each analyte

Analyte	Diffusivity (cm^2^ s^−1^)	Flow rate (μL min^−1^)						Transition (μL min^−1^)
		50	100	250	500	1000	2000	
Gd^17^	5.3 × 10^−6^	273	547	1366	2733	**5465**	**10** **930**	914
Insulin^18^	1.5 × 10^−6^	965	1929	4823	**9646**	**19** **292**	**38** **583**	259
Myoglobin^19^	1.13 × 10^−6^	1280	2561	**6402**	**12** **804**	**25** **608**	**51** **216**	195
HSA^18^	6.1 × 10^−7^	2372	4744	**11** **860**	**23** **719**	**47** **438**	**94** **876**	105

### 3.3. Matrix cleanup

The gadolinium recovery was 87 ± 4% when loading the sample under SPE conditions, and 92 ± 5% when loading the sample under TFC conditions. A one-tail *t*-test revealed that the TFC conditions provide significantly higher Gd concentrations than in the SPE (*N* = 3, *p* = 0.030). Humic and fulvic matter at a pH of 2 are protonated and bear no charge, do not complex Gd, but will bind to the sorbent through reversed phase interactions under SPE conditions, competing with the Gd and thus decreasing the sorption capacity for the latter. Moreover, the organic matter that is bound and could be eluted along with the Gd would provoke an ionization suppression in the ICP-MS, decreasing transiently the Gd sensitivity. The loading under TFC conditions excludes the organic matter from the resin, cancelling both the described effects and increasing the recovery.

### 3.4. Proof of concept: application to natural samples

The analysis of the raw river water yielded a free Gd concentration of 97 ± 2 ng L^−1^ and 92 ± 6 ng L^−1^ (*N* = 3) when processed under SPE and TFC conditions, respectively. A *t*-test reveals no significant differences between both magnitudes (*p* = 0.269). The complex with HMWC can probably be considered labile when the strong cation exchange resin competes for the polarizing Gd^3+^. The acidified fraction was 268 ± 3 ng L^−1^ (*N* = 3), and thus, through mass balance, the complexed fraction would be 176 ± 10 ng L^−1^. The flexibility demonstrated by our platform, allowing different SPE and TFC conditions, as well as acidifying the sample in-valve is unmatched by other standalone sample preparation techniques.

## 4. Conclusions

We have presented an unattended platform for performing TFC with off-the-shelf components, including the solid phase renewal as a front end to ICP-MS. TFC was previously only applied with dedicated expensive hardware, so we think that this contribution will help in spreading this powerful technique, par excellence, by integrating it in -omic workflows. The herein used system has a small footprint and is composed solely of a bidirectional pump able to deliver <100 bar and a stream selector, but it can also be implemented in most autosamplers. The most important benefits are the reduced use of sorbent and the truly unattended operation.

We have assessed the performance of TFC in terms of recovery of analytes and interference as a function of their MW and loading flow rates and finally, as a proof of concept, we have demonstrated the flexible performance of the BI-TFC platform by speciating Gd as a model analyte in surface water through different SPE and TFC approaches prior to ICP-MS. We also proved that the TFC conditions extend the sorbent life since the HMWC are not allowed to interact with it. The sample injection takes 12.6 s, which is shorter than the time required for analytical signal acquisition, allowing ultimate throughput through interleaving. The sorbent renewal takes 144 s, which is longer than the time required for data acquisition and thus discommended for online hyphenation to ICP-MS because of the extended argon consumption and system drift. LC-MS systems would not suffer from this drawback since the analysis time is orders of magnitude longer than the proposed sample preparation duration, and online hyphenation through the injector is readily available. We are currently working on a hardware rearrangement to unpack the sorbent with positive pressure for the sake of a faster performance and cleaning the frit from sample to sample, allowing its use as a renewable filter for the injection of raw environmental or clinical samples, such as raw blood in a metabolomic or metallomic analysis.

## Conflicts of interest

There are no conflicts of interest to declare.

## Supplementary Material

JA-036-D1JA00252J-s001

JA-036-D1JA00252J-s002

## References

[cit1] Herman J., Edge T., Majors R. (2012). LCGC North Am..

[cit2] Division of applications 08/974336, 2000, 1–29

[cit3] Couchman L. (2012). Biomed. Chromatogr..

[cit4] Koellensperger G., Galanski M., Keppler B. K., Hann S. (2016). J. Anal. At. Spectrom..

[cit5] Barreiro J. C., Luiz A. L., Maciel S. C. F., Maciel E. V. S., Lanças F. M. (2015). J. Sep. Sci..

[cit6] Herman J. L. (2005). Rapid Commun. Mass Spectrom..

[cit7] Miró M., Hartwell S. K., Jakmunee J., Grudpan K., Hansen E. H. (2008). Trends Anal. Chem..

[cit8] Wang J., Hansen E. H. (2003). Trends Anal. Chem..

[cit9] Cocovi-Solberg D. J., Rosende M., Michalec M., Miró M. (2019). Anal. Chem..

[cit10] Cocovi-Solberg D. J., Miró M. (2015). Anal. Bioanal. Chem..

[cit11] CocoSoft, https://sites.google.com/view/cocovisolberglab/cocosoft, accessed 5 February 2021

[cit12] Flow Injection Tutorial, https://www.flowinjectiontutorial.com/Methods3.0BeadInjection.html, accessed 15 June 2020

[cit13] Raju C. S. K., Lück D., Scharf H., Jakubowski N., Panne U. (2010). J. Anal. At. Spectrom..

[cit14] Rogowska J., Olkowska E., Ratajczyk W., Wolska L. (2018). Environ. Toxicol. Chem..

[cit15] Rudewicz P. J. (2011). Bioanalysis.

[cit16] World Intellectual Property Organization, WO199716724, 1997

[cit17] Fourest B. B., Duplessis J., David F. (1984). Radiochim. Acta.

[cit18] Vogel S., Life's Devices – The Physical World of Animals and Plants, Princeton University Press, 1984

[cit19] Tyn M. T., Gusek T. W. (1990). Biotechnol. Bioeng..

