# Supplementary material for: Sample preparation under turbulent flow with renewable sorbent
Source: J Anal At Spectrom. 2021 Oct 7;36(11):2306–11. doi: 10.1039/d1ja00252j (PMC8565614; doi:10.1039/d1ja00252j)
Supplement: JA-036-D1JA00252J-s002 [file JA-036-D1JA00252J-s002.pdf]

Electronic Supplementary Material (ESI) for Journal of Analytical Atomic Spectrometry.
This journal is © The Royal Society of Chemistry 2021

Suplementary information

Method example: Table SI1

Example of control method used for the natural sample analysis, as written in the CocoSoft control software. While the original code is readable, the right column provides comments and explanations.

Method	Description
Injector.load()<br>Plot(y=[[M6HP.pressure()]], title='Pressure', ylabel='bar', xlabel='seconds', hold=True, timeout=0.5)<br><br>DESIRED_MASS=4<br>HEARTCUT=70-(DESIRED_MASS-4)/1.10<br>LOADING_FLOW=1000<br>ACCELERATION=14800<br>SAMPLE_VOLUME=1000<br><br>AIR=10<br>CUP=1<br>HNO3=4<br>HNO3_6M=8<br>METHANOL=9<br>SAMPLE=5<br>SORBENT=2<br>TRANSFER=6<br>WASTE=7	Injector is set to load position; pressure is recorded every 0.5 s and several variables that will make the method more readable are initialized
Routine_define('Measure pressure')<br>Selector.position(TRANSFER)<br>M6HP.flow rate_uL_min(1000)<br>M6HP.dispense_uL()<br>P=0<br>Wait(5)<br>Loop(20)<br>P=max(P,M6HP.pressure())<br>Loop_end()<br>M6HP.flow rate_uL_min(0)<br>Wait(5)<br>Routine_end()	Routine for measuring the pressure.<br>The transfer line is perfused at 1 mL min^{-1} during ca. 14 seconds. After 5 seconds for stabilization, the maximum pressure is returned as P
Selector.position(AIR)<br>M6HP.aspirate_uL(10)<br>Loop(SAMPLE_VOLUME%100)<br>Selector.position(SAMPLE)<br>M6HP.aspirate_uL(100)<br>Selector.position(HNO3_6M)<br>M6HP.aspirate_uL(6)<br>Selector.position(CUP)<br>M6HP.dispense_uL(106)<br>Loop_end()<br>Selector.position(WASTE)<br>M6HP.dispense_uL(50)	Acidification. The desired volume of sample is transferred to the mixing cup in aliquots of 100 µL bracketed by HNO_3 6M.

Routine_call('Measure pressure')<br>PMAX = P<br>DESIRED_P = (DESIRED_MASS-0.1532) / 2.7187<br>While(PMAX<DESIRED_P*0.9)<br>M6HP.flow rate_uL_min(1000)<br>Selector.position(AIR)<br>M6HP.aspirate_uL(5)<br>Selector.position(METHANOL)<br>M6HP.aspirate_uL(20)<br>Selector.position(SORBENT)<br>M6HP.aspirate_uL((DESIRED_P - P)*1.62)<br>Wait(1)<br>Selector.position(TRANSFER)<br>M6HP.dispense_uL(100)<br>Routine_call('Measure pressure')<br>PMAX=P<br>While_end()	Packing the SPE µcolumn. The sorbent is aspirated bracketed with air and methanol and packed at 1 mL min^{-1}. The so formed column is conditioned with carrier. Pressure is measured. The procedure is repeated until at least 90% of the desired sorbent mass has been packed.
LOADING_FLOW=1000<br>Routine_call('Injection')<br>LOADING_FLOW=100<br>Routine_call('Injection')<br>LOADING_FLOW=1000<br>SAMPLE=CUP<br>SAMPLE_VOLUME*=1.1<br>Routine_call('Injection')	The sample is loaded by calling the main routine, 'Injection', under TFC conditions. Afterwards, the sample is processed again but at SPE flow rate. Finally, the acidified sample that was parked in the cup (port=1), is processed also by the same method at TFC flow rate.
Routine_define('Injection')<br>M6HP.flow rate_uL_min(200)<br>Selector.position(WASTE)<br>M6HP.dispense_uL(40)<br>ACCELERATION_VOL=(LOADING_FLOW**2)/<br>ACCELERATION/2<br><br>Loop(SAMPLE_VOLUME%100)<br>M6HP.flow rate_uL_min(1000)<br>Selector.position(SAMPLE)<br>M6HP.aspirate_uL(100)<br>Selector.position(WASTE)<br>M6HP.aspirate_uL(ACCELERATION_VOL)<br>Selector.position(TRANSFER)<br>M6HP.flow rate_uL_min(LOADING_FLOW)<br>M6HP.set_acceleration_uL_min2(ACCELERATION)<br>M6HP.dispense_uL(HEARTCUT+SAMPLE_VOLUME*<br>ACCELERATION_VOL)<br>M6HP.set_acceleration_uL_min2()<br>Loop_end()<br><br>M6HP.flow rate_uL_min(1000)<br>Selector.position(HNO3)<br>M6HP.aspirate_uL(100)	The sample is aspirated in 100 µL aliquots, followed by the acceleration compensation volume. They are dispensed to the SPE µ-column at the preselected loading flow rate, followed by an excess of carrier (water), which also helps cleaning the sorbent. After all the sample has been loaded, the analyte is eluted with a heartcut volume of HNO3 and then the sorbent cleaned and conditioned again with carrier.

Selector.position(TRANSFER)<br>M6HP.flow rate_uL_min(400)<br>Injector.load()<br>M6HP.dispense_uL(HEARTCUT)<br><br>Injector.inject()<br>M6HP.relay(1,0.5)<br><br>M6HP.dispense_uL(150-HEARTCUT)<br>Routine_end()	
M6HP.flow rate_uL_min(1000)<br>P=50<br>While(P>=1)<br>Selector.position(AIR)<br>M6HP.aspirate_uL(5)<br>Selector.position(METHANOL)<br>M6HP.aspirate_uL(40)<br>Selector.position(AIR)<br>M6HP.aspirate_uL(5)<br>Selector.position(TRANSFER)<br>M6HP.dispense_uL(45)<br>Loop(5)<br>M6HP.aspirate_uL(2)<br>Wait(1)<br>Loop_end()<br>Wait(5)<br>M6HP.flow rate_uL_min(200)<br>M6HP.aspirate_uL(60)<br>Selector.position(WASTE)<br>M6HP.flow rate_uL_min(1000)<br>M6HP.dispense_uL(200)<br>Routine_call('Measure pressure')<br>While_end()	Unpacking routine. The sorbent is wetted with a methanol plug, bracketed by air for preventing in-loop dispersion. 5 aspiration pulses of 2 µL allow to loosen the bed. Finally, it is aspirated into the loop at 1 mL min^{-1} and disposed to the waste port by flow reversal. If the pressure is higher than 1 absolute bar, the routine is repeated.
